# Hip dislocation during congenital short femur lengthening: a case report of successful conservative management

**DOI:** 10.11604/pamj.2025.50.20.46117

**Published:** 2025-01-10

**Authors:** Mohamed Achraf Ferjani, Mohamed Taghouti, Yosri Abcha, Almohimeed Abdullah, Mourad Jenzri, Khaled Kamoun

**Affiliations:** 1 Pediatric Orthopedic Department, Kassab Institute, El Manar University, Tunis, Tunisia,; 2 HafrAl-Batin Health Cluster, HafrAl-Baten, Saudi Arabia

**Keywords:** Child, femoral deficiency, limb lengthening, postoperative complications, case report

## Abstract

Congenital short femur (CSF) is a rare condition, often requiring iterative femoral lengthening to address limb length discrepancy (LLD). While effective, this procedure can be complicated by knee and hip instability and dislocation. We present the case of a 6-year-old boy with Kalamchi type I CSF and a predicted LLD of 12 cm who developed hip subluxation during progressive Orthofix-assisted femoral lengthening. Conservative management with closed reduction under sedation and pelvic cast immobilization was successful with a stable hip at 15 months follow-up with no stiffness or evidence of avascular necrosis. This case highlights the risk of hip subluxation during femoral lengthening for congenital short femur and demonstrates the effectiveness of conservative management in achieving successful results. Early recognition and prompt intervention are essential to prevent long-term complications.

## Introduction

Congenital short femur (CSF) is a rare disorder, often requiring iterative femoral lengthening to correct limb length discrepancy (LLD). This procedure, while effective, is not without risk. Serious complications can occur, including hip or knee subluxation or dislocation. Although previous reports have identified contributing factors to hip instability during lengthening for CSF [1,2], the optimal management of established hip dislocation remains poorly defined [3,4]. This case report details the conservative management of a hip dislocation complicating femoral lengthening in a child with CSF and highlights the successful outcome achieved with this approach.

## Patient and observation

**Patient information:** a 6-year-old boy with no past medical history presented to the outpatient department with right lower limb shortening noticed since birth.

**Clinical findings:** in clinical exam, the right femur was notably shorter, though the ipsilateral hip and knee maintained a full range of motion. There was no history of hip dislocation, and Lachman and drawer tests confirmed the absence of laxity.

**Timeline of current episode:** right lower limb shortening was noted at birth. At the initial visit, examination, and radiographs led to a diagnosis of Kalamchi type I congenital short femur (CSF) with a predicted 12cm LLD. In April 2021, telescopic nailing was performed before lengthening. Femoral lengthening was initiated using an Orthofix external fixator at a rate of 1 mm/day. One month post-operatively, the patient developed hip pain, adduction, and subluxation, prompting a halt to the lengthening. Closed reduction was performed under anesthesia, and a spica cast was applied. The cast was removed after two months and replaced with a broomstick abduction brace for an additional two months. At the 15-month follow-up, the hip was stable, fully mobile, and showed no signs of avascular necrosis.

**Diagnostic assessment:** initial evaluation included a clinical examination demonstrating a right femur shortening compared to the left, with full range of motion in the hip and knee, and no hip instability. Lower limb radiographs (teleroentgenogram) confirmed the shortening, showing a 7cm discrepancy in the right femur with no leg length discrepancy or other apparent congenital abnormalities. The affected hip had an acetabular index of 21° and a center-edge angle of 20°. The contralateral hip had an acetabular index of 17° and a center-edge angle of 23°. The femoral head version was neutral. The neck-shaft angle was 130°. The hip joint was congruous preoperatively. The projected LLD at maturity was calculated to be 12cm using the multiplier method [5]. Following the onset of hip pain and adduction one month after lengthening initiation, radiographic evaluation revealed hip subluxation. Examination under anesthesia confirmed a reducible and congruent hip.

**Diagnosis:** the final diagnosis was hip subluxation secondary to femoral lengthening in a patient with Kalamchi type I congenital short femur (CSF). Other diagnoses considered, before the onset of hip pain and adduction, included simple pain related to the lengthening procedure.

**Figure 1:**
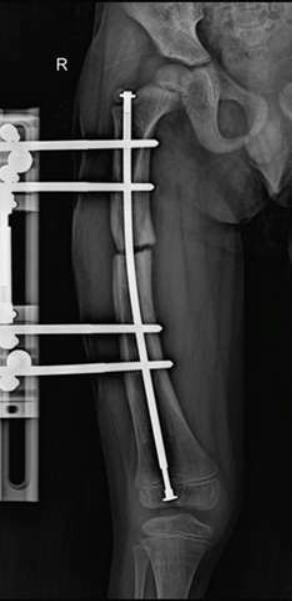
anteroposterior femur X-ra —setting up the Orthofix external fixator with the telescopic nail

**Figure 2:**
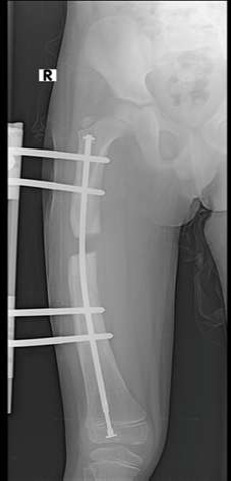
anteroposterior femur X-ray—start of distraction of the femur

**Figure 3:**
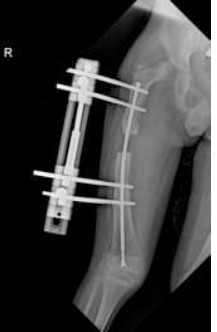
anteroposterior hip X-ray—excentration of the femoral head following distraction

**Figure 4:**
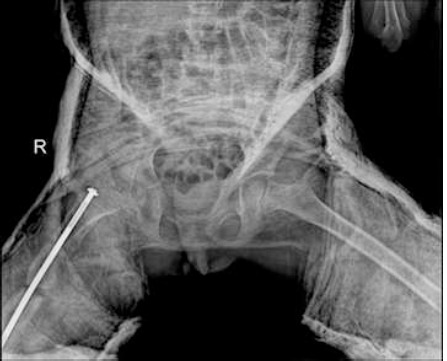
anteroposterior hip X-ray—after hip reduction under anaesthetic in the cast

**Figure 5:**
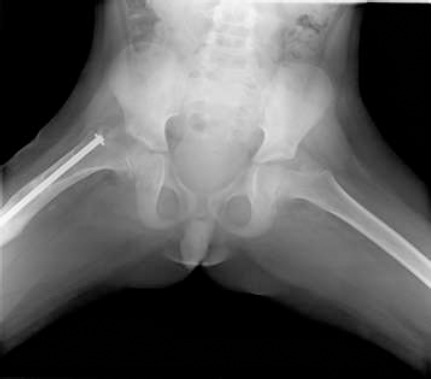
anteroposterior hip X-ray—right hip is in place at cast removal

**Therapeutic interventions:** initial treatment consisted of surgical intervention for femoral lengthening using an Orthofix external fixator (Figure 1), preceded by telescopic nailing for stabilization. Lengthening was performed at a rate of 1 mm/day, beginning 10 days post-osteotomy (Figure 2). One month after lengthening was initiated, the patient developed hip subluxation (Figure 3). In response to this complication, the lengthening process was temporarily halted. Subsequently, closed reduction of the hip was performed under general anesthesia. This was followed by immobilization in a spica cast for two months (Figure 4). After cast removal, the patient transitioned to broomstick abduction bracing for an additional two months to maintain hip stability (Figure 5).

**Figure 6:**
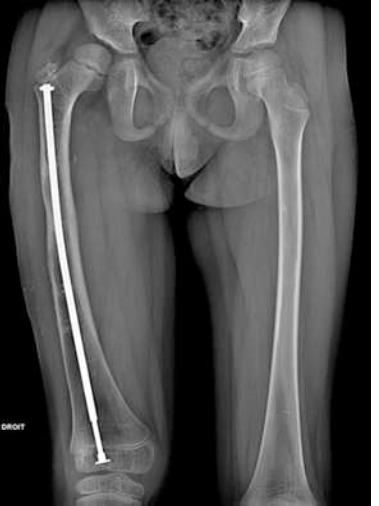
anteroposterior hip X-ray—reduced hip with no signs of avascular necrosis of the head at last follow-up

Follow-up and outcome of interventions: at the 15-month follow-up appointment, the patient demonstrated a stable hip with the full range of motion and no signs of avascular necrosis (Figure 6). The child remains asymptomatic and in excellent clinical condition, with no reported recurrence.

**Patient perspective:** “The cast was really uncomfortable, and I didn't like not being able to move around much. I'm glad my hip doesn't hurt anymore, and I can play with my friends again.”

**Informed consent:** the patient´s parents gave informed consent for using the data file for scientific publication. Authors certified that their child couldn't be recognized in the clinical photo.

## Discussion

While limb lengthening procedures carry inherent risks, the complication rate is notably higher in patients with CSF compared to other conditions [4]. Outcomes are further influenced by the presence of other associated congenital anomalies [4]. Hip subluxation or dislocation during femoral lengthening arises from biomechanical abnormalities that disrupt the balance of force vectors across the hip joint [6]. Predisposing factors in CSF include acetabular dysplasia, coxa vara, femoral head retroversion, and hip joint incongruity, all of which increase the susceptibility of the hip to posterior dislocation under heightened force vectors [6,7]. Even during progressive femoral lengthening, a longitudinal distraction force is transmitted to the acetabulum through the femoral head. In cases of acetabular dysplasia with inadequate lateral coverage, this force can result in hip subluxation or dislocation.

According to 0 *et al*. [4], an acetabular index of less than 25° and a center-edge (CE) angle greater than 20° are essential before initiating femoral lengthening to minimize the risk of hip dislocation. They further recommended addressing acetabular dysplasia before lengthening if the acetabular index exceeds 25° to help prevent hip subluxation. Suzuki *et al*. [6] reported that during femoral lengthening, hips with a CE angle greater than 20° did not experience complications, whereas those with a CE angle of 20° or less showed deterioration. They attributed this to altered force transmission, where the femoral head's load was directed away from the inner wall of the socket, leading to subluxation. They recommended that for hips with a preoperative CE angle of 20° or less, bony procedures such as innominate osteotomy should be performed before initiating femoral lengthening [6,8]. In our case, the affected side presented with an acetabular index of 21° and a CE angle of 20°, compared to 17° and 23°, respectively, on the unaffected side. The femoral head was in neutral version with the femoral condyles, indicating the absence of femoral neck anteversion, which was measured at 20° on the normal side. The neck-shaft angle was 130°, and the hip joint was congruous preoperatively.

When occurring, hip dislocation can be managed by open reduction or conservatively, as in our case where the reduction was done under anesthesia by manipulating the hip in progressive abduction and mild internal rotation. This conservative approach was prioritized, demonstrating its effectiveness in specific clinical contexts. Upon detecting hip subluxation early in the lengthening process, distraction was halted, and a closed reduction was attempted under general anesthesia. At the latest follow-up, this non-invasive strategy resulted in a stable, congruous hip with full mobility. This approach highlights the importance of timely detection and intervention, emphasizing that conservative measures, when applied appropriately, can achieve favorable outcomes without necessitating invasive procedures.

## Conclusion

In conclusion, this case report demonstrates that conservative management can be an effective approach to managing hip dislocation during femoral lengthening in congenital short femur cases. Despite the complexity and potential risks associated with lengthening procedures, such as hip dislocation, our conservative strategy led to a successful outcome with full hip mobility and stability at follow-up. This highlights the importance of timely intervention, careful monitoring, and the potential for non-invasive treatment options, especially in cases with less severe instability. Further studies and case reports are needed to refine guidelines for managing hip subluxation in femoral lengthening procedures, but this case provides a valuable contribution to the broader understanding of conservative management in pediatric orthopedics.
